# Impairments in face discrimination and emotion recognition are related to aging and cognitive dysfunctions in Parkinson’s disease with dementia

**DOI:** 10.1038/s41598-020-61310-w

**Published:** 2020-03-09

**Authors:** Mary Wen-Reng Ho, Sarina Hui-Lin Chien, Ming-Kuei Lu, Jui-Cheng Chen, Yu Aoh, Chun-Ming Chen, Hsien-Yuan Lane, Chon-Haw Tsai

**Affiliations:** 1 Graduate Institute of Biomedical Sciences, China Medical University, Taichung, Taiwan; 2 College of Medicine, China Medical University, Taichung, Taiwan; 3 Department of Neurology, China Medical University Hospital, Taichung, Taiwan; 4 Department of Radiology, China Medical University Hospital, Taichung, Taiwan; 5 Department of Psychiatry, China Medical University Hospital, Taichung, Taiwan; 6 Department of Psychology, College of Medical and Health Sciences, Asia University, Taichung, Taiwan; 7 Department of Neurology, China Medical University Hsinchu Hospital, Hsinchu, Taiwan

**Keywords:** Dementia, Parkinson's disease

## Abstract

Patients with Parkinson’s disease (PD) suffer from motor and non-motor symptoms; 40% would develop dementia (PD-D). Impaired face and emotion processing in PD has been reported; however, the deficits of face processing in PD-D remain unclear. We investigated three essential aspects of face processing capacity in PD-D, and the associations between cognitive, neuropsychiatric assessments and task performances. Twenty-four PD-D patients (mean age: 74.0 ± 5.55) and eighteen age-matched healthy controls (HC) (mean age: 71.0 ± 6.20) received three computerized tasks, *morphing-face discrimination*, *dynamic facial emotion recognition*, and *expression imitation*. Compared to HC, PD-D patients had lower sensitivity *(d’)* and greater neural internal noises in discriminating faces; responded slower and had difficulties with negative emotions; imitated some expressions but with lower strength. Correlation analyses revealed that patients with advancing age, slow mentation, and poor cognition (but not motor symptoms) showed stronger deterioration in face perception. Importantly, these correlations were absent in the age-matched HC. The present study is among the first few examined face processing in patients with PD-D, and found consistent deficits correlated with advancing age and slow mentation. We propose that face discrimination task could be included as a potential test for the early detection of dementia in PD.

## Introduction

Parkinson’s disease (PD) affects 2–3% of the population worldwide over the age of 65^1^; about 40% of PD would develop dementia (PD-D)^2^. Motor dysfunctions are core clinical features in PD^1^; however, the contributions of non-motor symptoms to a reduced quality of life are also widely recognized^3^. Patients suffer from an array of non-motor symptoms including autonomic, digestive, cognitive, and affective dysfunctions, as well as disturbances in visual perception^4,5^. Among the visual disturbances, impairments in high-level perception such as facial identity and emotion processing drastically affect the patients’ social interactions with others^6,7^.

Successful recognition of facial identity and emotional expressions is fundamental to social life, the hallmark of human perceptual skills develop soon after birth^8,9^. Early studies reveal impaired face recognition in PD^10,11^, with performance correlated with gray matter density in the fusiform face areas (FFA)—the region involved in the visual analysis of face structure in the healthy brain^12,13^. Recent studies investigating face recognition in PD revealed memory deficits for both familiar and unfamiliar faces^14–16^. Cousin *et al*.^17^, reported that impairment in configural processing (also processed in the FFA) predicted unfamiliar face recognition deficits in PD patients. While most studies focused on recognition memory in PD patients, very few inspected their perceptual discriminability under the framework of the signal detection theory^18^; it is unclear whether the patient’s impairments in recognition memory reported previously reflects an elevated discrimination threshold for processing faces, or greater internal neural noises resulting in less efficient perceptual decision making.

Patients with PD were known to have difficulties in emotion recognition and expression production. Expression identification tasks using static emotional stimuli revealed marked deficits in recognizing negative emotions (i.e., Anger, Disgust, and Fear)^19–21^. However, it has been pointed out that recognizing static facial expression requires imagery of the motor pattern in each emotion^21^. Kan *et al*.^21^ adopted dynamic facial expression stimuli (i.e., adding cues of motor imageries) and reported improved performance with dynamic stimuli than static ones in PD patients. For expression production, one notable symptom of PD is hypomimia^22^, a decrease in facial expressiveness^4^. Using facial electromyography, Livingstone *et al*.^23^ found that patients with PD had lower amplitude and delayed onset of expression. Other studies using behavioral measures showed poor performance in imitating facial expressions in PD rated by human observers^23–25^. A recent study with Chinese patients^24^ supported the mirror neuron hypothesis^25^; the PD group scored lower on both facial and vocal emotion processing than the healthy controls^25^ and exhibited a significant correlation between emotion recognition and expression performance^24,25^.

Advancing age influences the clinical progression of PD and associates with the development of dementia^26^. PD dementia (PDD) has a unique clinical profile and neuropathology, distinct from Alzheimer’s disease (AD)–another major cause of dementia. Although pathologically different, both PD and AD are associated with the locus coeruleus (LC: the major noradrenergic nucleus in the brain) degeneration^27–29^. In a recent review, Peterson and Li (2018)^30^ found that LC degeneration changes the connectivity accompanying deficient capacity in suppressing default mode network (DMN) activity and increasing saliency and task control network activities to meet behavioral challenges, supporting the proposition that noradrenergic dysfunction contributes to memory and cognitive impairment in AD and PD.

**Figure 1:**
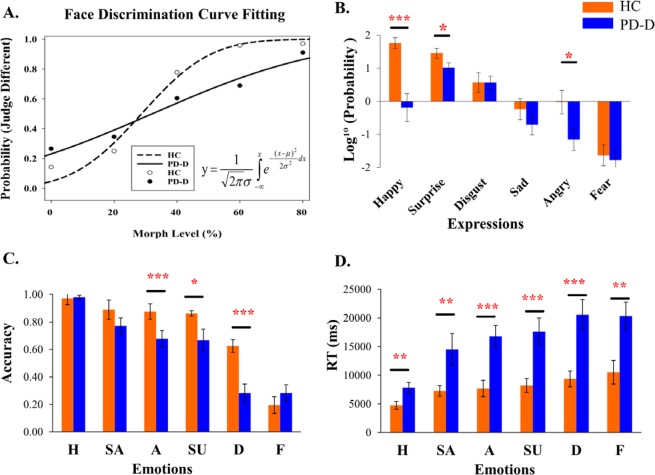
The main results of the three tasks. The group mean fitted curves in the Face Discrimination task (HC: open circle, PD-D: solid circle) (Panel A). The group mean log probabilities of Expression Imitation task (Panel B). The orange and blue bars represent the HC and the PD-D groups, respectively. The group mean accuracies (Panel C) and response times (Panel D) of the Dynamic Emotion Recognition task. (H = happy, SA = sad, A = anger, SU = surprise, D = disgust, and F = fear) (*p < 0.05, **p < 0.01, ***p < 0.001).

Concerning face perception, impaired face memory and emotion expression in PD has been well-documented; however, the nature and extent of face-processing deficits in PD-D remain unclear. Therefore, the present study investigated face processing capacity in patients with PD-D with three computerized tasks focusing on three essential aspects: face discrimination, emotion recognition, and expression imitation (See Fig. 1). The first task was *Morphing Face Discrimination*, where we examined how well can PD-D patients distinguish two morphing face images of subtle changes using signal detection theory^18^ and threshold estimation (i.e., finding the smallest physical change that the participant needed to detect the differences). The second task was *Dynamic Facial Emotion Recognition*, where participants were to identify the six basic emotions from neutral to fully expressed state. The third task was *Expression Imitation*, where participants imitated the six basic expressions and were scored by an expression coding software, the iMotions^TM^ Affectiva. We also conducted correlation analyses to reveal the pattern of associations between the participant’s age, cognitive, psychiatric and clinical assessments (MMSE, ADAS-cog, HAM-D, UPDRS) and their task performances.

## Results

### Morphing face discrimination

In the morphing face discrimination task, half of the trials were physically the same (the 0% morph level) and half were different (all other morph levels); therefore, we adopted the signal detection theory (SDT)^18^ to code the responses into four categories: *hits*, *miss*, *false alarm*, *correct rejection*, and calculated the perceptual sensitivity index d’, defined as [Z(Hit)-Z(False Alarm)] (See the bottom half of Table 1). We defined “Hit” as responded as “different’ when the comparison face was physically different (“Hit” was computed by averaging across the responses at the 20%, 40%, 60%, and 80% morph levels). “Miss” was responded as “same” when the comparison was physically different (“Miss” was also computed by averaging across the 20%, 40%, 60%, and 80% morph levels). “False Alarm” was responded as “different” when the comparison was physically the same, and “Correct Rejection” was responded as “same” when the comparison was the same (the computation of “False Alarm” and “Correct Rejection” involved only the 0% morph level). The PD-D group had a significantly greater *false alarm* at the 0% morph level than the HC group (*p* = 0.03), indicating that the patients tended to misjudged the physically same face as a “different” face stimuli. For the discriminability or sensitivity index d’, the PD-D group had a significantly lower d’ than the HC group (*p* = 0.004).

**Table 1:** Participants’ demographics and characteristics (Means and SD).

Characteristics	PD-D (n = 24)	HC (n = 18)	*P*-value
Gender	F: 11 M: 13	F: 10 M: 8	0.53
Age Range	62~81	63~81	
Age Mean	74.0 ± 5.55	71.0 ± 6.20	0.11
MMSE	19.88 ± 4.14	29.28 ± 1.07	<001
HAM-D	5.29 ± 3.17	0.61 ± 1.09	<001
ADAS-Cog	18.64 ± 11.46	2.85 ± 1.47	<001
UPDRS (total)	48.91 ± 25.09	N/A	
UPDRS I	4.17 ± 2.61	N/A	
UPDRS II	12.52 ± 7.69	N/A	
UPDRS III	32.22 ± 16.93	N/A	
Face Discrimination (Mean and SE)			
Hit	0.619 ± 0.05	0.735 ± 0.03	0.06
Miss	0.381 ± 0.05	0.265 ± 0.03	0.06
Correct Reject	0.734 ± 0.05	0.858 ± 0.030	0.04
False Alarm	0.275 ± 0.05	0.149 ± 0.03	0.03
*d’* (d-prime)	1.192 ± 0.16	1.848 ± 0.15	0.004
*µ* (Threshold)	32.08% ± 3.25%	28.73% ± 2.40%	
*σ* (1/slope)	0.436 ± 0.05	0.171 ± 0.03	

UPDRS: Unified Parkinson’s disease Rating Scale.

MMSE: Mini-Mental State Examination.

HAM-D: Hamilton Depression Rating Scale.

ADAS-Cog: Alzheimer’s Disease Assessment Scale Cognitive Behavior Section.

Moreover, considering the morph level of the face stimuli is a continuous variable, we further fitted the group psychometric functions with a *normal cumulative distribution* model to estimate the *discrimination threshold (μ)* and the *slope* parameter (σ) for each group (See bottom half of Table 1). The PD-D group’s *threshold (μ)* was at 32.8%, and the HC group was at 28.73%. To test whether the *thresholds* of the two groups were different, we used a 95% confidence interval estimation^31^ (*μ*_*HC*_ ± 1.96*SE_*HC*_) based on the HC group (See Fig. 1A). This was because we consider HC as the baseline to be compared with the PD-D group (H_0:_ *μ*_HC_ = *μ*_PD-D_). The lower limit of the *μ*_*HC*_ was 24.02%, the upper limit was 33.43%; the threshold of the PD-D group (32.08%) fell within the confidence interval; hence the thresholds of both groups were not different. Likewise, to test whether the *slope parameters* of the two groups were different, we used a 95% confidence interval estimation^31^ (*σ*_HC_ ± 1.96*SE_*HC*_) based on the HC group. The HC had a lower limit of 0.11, an upper limit of 0.23, and the slope parameter of PD-D group was higher than the upper limit (0.436 > 0.23). Hence, the PD-D group psychometric function was significantly shallower than that of the HC group, indicating that patients with PD-D had greater neural internal noises and were more uncertain in discriminating faces.

### Dynamic facial emotion recognition

We conducted two 2-way mixed ANOVAs on accuracy and response time separately, with *Group* as the between-subject factor, and *Emotion Type* as the within-subject factor. For accuracy, the *Group* main effect was significant, (*F* (1,40) = 11.077, *p* = 0.002, η^2^ = 0.217.); the HC group had a higher overall accuracy (*M* = 0.736, *SE* = 0.019) than the PD-D group (*M* = 0.609, *SE* = 0.030). The main effect of *Emotion Type* was significant (*F* (5, 200) = 45.719, *p* < 0.001, η^2^ = 0.553). From high to low, the mean accuracies for Happy, Sad, Anger, Suprise, Disgust, and Fear were 0.976 (*SE* = 0.012), 0.830 (*SE* = 0.039), 0.776 (*SE* = 0.040), 0.764 (*SE* = 0.054), 0.453 (*SE* = 0.049), and 0.238 (*SE* = 0.043), respectively. Importantly, the Group * Emotion Type interaction was significant, (*F* (5, 200) = 3.688, *p* = 0.003, η^2^ = 0.084). We further analyzed the *Group* simple main effect for each emotion (Fig. 1C). With an adjusted error rate at *α* level = 0.05/6 = 0.008, the HC group performed significantly better in perceiving Anger (*t*(40) = 2.633, *p* = 0.006), Disgust (*t*(40) = 3.550, *p* < 0.001); and marginally better for Sad (*p* = 0.05), and Surprise (*p* = 0.03) than the PD-D group.

For response time (RT), three participants’ data from the PD-D group were excluded because they took longer than 1-minute to respond. The *Group* main effect was significant (*F* (1,37) = 18.754, *p* < 0.001, η^2^ = 0.336.), the PD-D group (*M* = 15080 ms, *SE* = 1114) responded markedly slower than the HC group (*M* = 7970 ms, *SE* = 1240). The *Emotion Type* main effect was significant (*F* (5,185) = 10.097, *p* < 0.001, η^2^ = 0.214). From fast to slow, the mean RT for Happy, Sad, Anger, Suprise, Fear and Disgust were 5968 ms (*SE* = 592.9), 9396 ms (*SE* = 1074.0), 11840 ms (*SE* = 1209.9), 13020 ms (*SE* = 1464.5), 13940 ms (*SE* = 1403.6), and 14970 ms (*SE* = 1564.4), respectively. The Group * Emotion Type interaction was significant, (*F* (5, 185) = 2.470, *p* = 0.034, η^2^ = 0.063)(Fig. 1D), thus we further analyzed the *Group* simple main effect for each emotion. With an adjusted error rate at *α* level = 0.05/6 = 0.008, the HC group were significantly faster in recognizing Happy (*t*(37) = 2.714, *p* = 0.004), Anger (*t*(37) = 3.820, *p* < 0.001), Suprise (*t* (37) = 3.563, *p* = 0.001), Fear (*t* (37) = 3.077, *p* = 0.003), and Disgust (*t* (37) = 3.754, *p* < 0.001).

### Expression imitation

Each participant’s imitation performance was first analyzed by the iMotions Affectiva software, which detects facial landmarks and classifies facial expressions in return with numeric output scores for facial expressions. The numerical scores were values between 0 (no expression) to 100 (expression fully present). Because the range of individuals’ output scores for different expressions expanded over 6 log units; therefore, we applied logarithm transformation (log of 10). We then conducted a 2-way mixed ANOVA with *Group* and *Expression Type* on the log expressive scores. The *Group* main effect was significant (*F* (1,38) = 12.997, *p* = 0.001, η^2^ = 0.255), the HC group had a greater mean log expressive score (*M* = 0.314, *SE* = 0.144) than the PD-D group (*M* = −0.372, *SE* = 0.124). The *Expression Type* main effect was significant (*F* (5,190) = 29.643, *p* < 0.001, η^2^ = 0.438), the Group * Expression Type interaction was significant (*F* (5,190) = 3.323, *p* = 0.007, η^2^ = 0.080) (see Fig. 1B). With the adjusted *α* level (0.05/6 = 0.008), the HC group showed a higher log expressive score in imitating Happy (*t*(38) = 2.179, *p* < 0.001), and marginally higher for Anger (*p* = 0.039), and Surprise (*p* = 0.062) than the PD-D group.

### Correlations among age, the clinical assessments, and the tasks

We conducted Pearson’s correlations to explore the associations among the participant’s age, clinical assessments (MMSE, ADAS-cog, and UPDRS-I) and their task performances (d’ of the Face Discrimination, RT for Dynamic Emotion recognition, and log probability of Expression Imitation) for each group. Table 2 summarizes the correlation strengths and p-values. With the adjusted *α* level (0.05/10 = 0.005), none of the correlations reached statistical significance for the HC group (lower panel of Table 2). Only the MMSE showed a marginally negative correlation with emotion recognition RT (*r* = −0.535, *p* = 0.022), indicating that individuals with higher scores tended to recognize emotions faster. With the adjusted *α* level (0.05/15 = 0.003), the PD-D group exhibited certain noteworthy trends or correlations (upper panel of Table 2). First of all, Age marginally correlated with ADAS-Cog (*r* = 0.456, *p* = 0.029) and *d*’ (*r* = −0.433, *p* = 0.039), indicating that advancing Age worsened cognitive functions and face discrimination. UPDRS subscores I (mentation, behavior and mood) correlated with MMSE (*r* = −0.661, *p* = 0.001), RT (*r* = 0.696, *p* < 0.001), and marginally with *d’* (*r* = −0.451, *p* = 0.035). MMSE scores negatively correlated with RT (*r* = −0.534, *p* = 0.007). The ADAS-Cog scores marginally correlated with d’ (*r* = −0.418, *p* = 0.042) and RT(*r* = 0.433, *p* = 0.039). These indicated that patients with slow mentation or poor cognition tended to show stronger deterioration in face discrimination and were slower in emotion recognition. Lastly, patient’s *d*’ in face discrimination marginally correlated with RT in emotion recognition (*r* = −0.482, *p* = 0.020).

**Table 2:** Correlations and P-values (in parenthesis) of both groups.

		Age	Assessment		Task 1	Task 2	Task 3
			MMSE	ADAS-Cog	D-prime	RT	Imitation
PD-D^a^	UPDRS I^c^	0.280 (0.195)	−0.661 (0.001)**	0.426 (0.043)^†^	−0.451 (0.035)^†^	0.691 (<0.001)***	0.148 (0.512)
	MMSE	−0.397 (0.055)	1	−0.671 (<0.001)***	0.345 (0.107)	−0.534 (0.007)^††^	0.058 (0.792)
	ADAS-Cog	0.456 (0.029)^†^	—	1	−0.453 (0.035)^†^	0.433 (0.039)^†^	0.111 (0.623)
	D-prime	−0.433 (0.039)^†^	—	—	1	−0.482 (0.020)^†^	0.303 (0.171)
	RT	0.369 (0.076)	—	—	—	1	0.040 (0.855)
	Imitation	−0.013 (0.952)	—	—	—	—	1
HC^b^	MMSE	0.096 (0.703)	1	−0.394 (0.105)	0.038 (0.879)	0.535 (0.022)^†^	0.313 (0.221)
	ADAS-Cog	0.297 (0.231)	—	1	−0.135 (0.593)	0.027 (0.915)	0.295 (0.251)
	D-prime	−0.164 (0.515)	—	—	1	0.366 (0.135)	0.320 (0.210)
	RT	0.001 (0.997)	—	—	—	1	0.339 (0.184)
	Imitation	−0.137 (0.601)	—	—	—	—	1

Task 1: Morphing face discrimination D-prime = SDT Sensitivity Index.

Task 2: Emotion recognition; RT = response time.

Task 3: Expression imitation log probability.

(^†^p < 0.05, ^††^p < 0.01, **p = 0.001, ***p < 0.001).

^a^Parkinson’s disease with dementia.

^b^Healthy control.

^c^Non-motor function (mentation, behavior, and mood).

## Discussions

The present study explored three essential aspects of face perception in patients with Parkinson’s disease who developed dementia (PD-D) and in age-matched healthy adults as a control group. We used SDT and curve-fitting procedures to analyze both groups’ perceptual sensitivity with morphing face discrimination. The PD-D group tended to misjudge same faces as different, had a lower sensitivity (d’) and a shallower function in discriminating faces. For dynamic facial emotion recognition, the PD-D group had greater difficulties recognizing negative emotions and were significantly slower than HC group. Lastly, overall the PD-D group performed worse in imitating facial emotions but could imitate some expressions. Additionally, we found consistent deficits in face and emotion processing that correlated with advancing age, slow mentation, and poor cognitive functions in the PD-D group. In contrast, the HC group did not exhibit declines in face and emotion processing and their performances did not correlate with advancing age; meaning that advancing age is not a critical determinant for the age-matched healthy adults, but it is a critical determinant of clinical progression in the PD-D group.

In the morphing face discrimination task, detecting subtle changes in morphing stimuli requires a mixture of the featural and configural processing (especially the holistic processing–glues all features together)^32,33^. Rossion, (2013)^34^ had explained judging whether two faces are the same engages holistic processing that requires fixating on all cues across the entire face; therefore, the higher *False Alarm* in PD-D suggested their difficulties in using configural processing to analyze unfamiliar faces. Our finding is consistent with Cousin *et al*.^17^ reporting a weaker configural processing in PD patients. Moreover, as Parkinsonism progressed to the demented stage^35^, it may lead to higher neural internal noises that are inherent to sensory neurons as a limiting factor in signal transduction^36^, which was observed in the shallower psychometric function of the PD-D patients. It is convincible that the greater internal noises (i.e., less efficient perceptual decision) may contribute to the poor performance on face recognition deficits reported previously^14–17^. Importantly, the link between the task performances (d’ and RT), cognitive and psychiatric assessments (MMSE, ADAS-Cog, and HAM-D), and non-motor functions of UPDRS I in the PD-D group were significantly correlated, meaning that patients with better cognitive functions maintain better face discrimination and faster to recognize emotions, and vice versa. Our findings agree with studies reporting that patients with Alzheimer’s Disease (AD) were impaired in discriminating facial identities and in naming emotions and that AD patients’ deficits of facial discrimination and emotion naming correlated with the MMSE and Raven scores^37^. Notably in our study, the visual-spatial subcomponent of MMSE (copying intersecting pentagons) and ADAS-Cog (figure drawing) correlated with d’ and FA in the PD-D patients, affirming that disturbed visuospatial construction skills were associated with impaired face discrimination^6^.

In the dynamic emotion recognition task, overall the PD-D group was able to answer significantly better than the chance level (1/6), indicating their ability to recognize dynamic facial emotions is moderately intact, which supported the studies showing better emotion recognition performance with dynamic facial stimuli in PD patients^21,38^. Importantly, PD-D also struggled more with negative emotions and tended to falsely identify Disgust as Anger, or Fear as Surprise, similar to the deficits reported in PD^19–21^. Our findings indicate that impairment of recognizing negative emotions persisted from PD to the PD-D stage; and may be explained by the observation that older adults tend to focus more on the mouth region (i.e., less diagnostic in identifying negative emotions) and less on the eyes^39,40^. For the expression imitation task, the PD-D group performed significantly worse in imitating *Happy*, consistent with Livingstone *et al*.^23^ who reported that PD patients had little or no reaction in their zygomaticus major muscle region after presenting the emotion *Happy*. PD patients suffered from dysphagia due to bradykinesia^1^ that involved the oro-buccal region^41^, which may pertain to why it was difficult for the PD-D group to imitate *Happy*. Moreover, imitation involves motor imagery. It is reported that patients with putamen lesions were impaired with motor imagery^42^, suggesting that the basal ganglia play an important role in motor imagery. Hence, the loss of dopaminergic neurons of the substantia nigra seen in PD-D affects the basal ganglia; this, in return, could lead to motor imagery impairment. Notably, in the present study, the link between emotion recognition and imitation was absent in both groups; therefore, not supporting the mirror-neuron hypothesis^24,25^. However, although disputable, the concept of motor theories of perception claims that motor processes play an essential role in perceiving actions^43^, but perception itself could be spared despite the impairments^44^. Another explanation is that the negative emotion imitation readings in the present study were scarcely detected by iMotions^TM^ Affectiva (perhaps due to cultural differences in expressing negative emotions^45^), lowering the correlation strength.

In conclusion, the present study was among the first few exploring three important aspects of face processing in patients with PD-D. Although face processing in PD-D is unequivocally worse than HC, their capacity is preserved at some level, indicating a partially intact core face perception system^13^. The empirical results reported in this study were subject to some limitations such as the lack of recruiting domestic participants to rate both groups’ expression imitation to validate the readings by iMotion Affectiva, and the response time in the dynamic emotion recognition task was not a direct measure. We are also aware that we did not compare PD-D and PD directly; however, our results in emotion recognition and expression imitation did not show much difference between them. Nevertheless, we discovered at the demented stage, the impairments in face and emotion processing seemed to correlate with advancing age, slow mentation, poor cognitive and visual-spatial functions, but not with motor symptoms. We propose that face discrimination could be included as a potential visual test for the early detection of dementia in PD.

## Methods

### Participants

Twenty-four patients with PD-D (11 women, mean age 74.0 ± 5.55) participated in the study. The sample size was predetermined by using GPower 3.1 calculation (alpha = 0.05, power = 0.80, with an effect size of 0.80 resulting in a sample size of 21)^46^. The diagnosis of PD-D was based on the criteria proposed by the 2007 movement disorders PD-D task force^47^. The core features include (i) diagnosis of PD according to the UK Brain Bank criteria^48^; and (ii) a dementia syndrome with insidious onset and slow progression developing within the context of PD. Patients did not have clinical conditions, such as systemic illness, vascular dementia, or drug intoxication. Only patients with Mini-Mental State Examination (MMSE) score between 10–26 were recruited. Additional inclusion criteria include (1) age between 50–90 years old, (2) laboratory assessments (including blood and biochemical tests) that were clinically insignificant, (3) at least six years of formal education or can communicate effectively and are capable of completing the assessments. Based on our previous report^49^, medications currently used by patients had to be stabilized for at least three months before inclusion and remain unchanged throughout the study period. The PD-D patients received the tasks in the morning while withheld the antiparkinsonian agents the night before. Two additional patients were tested but excluded because of inability to complete the experiment. The PD-D group received assessments of Unified Parkinson’s disease rating scale (UPDRS I~III), the Hamilton Depression Rating Scale (HAM-D), and the Alzheimer’s Disease Assessment Scale-Cog (ADAS-cog).

For the age-matched healthy controls (HC), eighteen adults (10 women, mean age 71.0 ± 6.20), with no history of neurological or mental illnesses, participated as the control group. Additional inclusion criteria (age, laboratory assessment, education) were the same as the PD-D group. The HC group also underwent cognitive and psychiatric assessments of MMSE (>26), HAM-D, and ADAS-Cog to rule out cognitive impairment and depression. All participants reported having a normal or self-supplied best-corrected vision (bifocals or corrected lenses) for the tests. We were aware that the sample size of HC group was smaller than that of PD-D group, however, it was difficult to find age- and gender-matched healthy participants as our control group who also met the inclusion criteria. Participants provided written, informed consent before the study. All methods conformed with relevant guidelines and regulations; the experimental protocols were approved by the Research Ethics Committee of China Medical University and Hospital Research Ethics Center, Taichung, Taiwan (the IRB certificate number: CMUH105-REC1-023). Table 1 summarizes the group characteristics and clinical assessments.

### Stimuli, apparatus, and procedures

Participants received (1) *Morphing Face Discrimination*, (2) *Dynamic Emotion Recognition*, and (3) *Expression Imitation task* in one visit. The first two tasks were run on a laptop computer (Acer eMachines E732) and with E-Prime Professional 2.0 (Psychological Software Tools, Sharpsburg, PA). The third task used a different laptop (Acer TravelMate P259) and with iMotions Affectiva (Version 4.0, Boston, USA).

#### Task 1: Morphing face discrimination

The morphing face stimuli and procedures were adapted from Chien *et al*.^32^. Two female (A/B) and two male faces (D/E) were selected (frontal view, neutral expression, oval-cropped, gray-scale). FantaMorph 5 Deluxe (Abrosoft Co. Nebraska, USA) was used to create two sets of morphing images of 20% intervals (0%, 20%, 40%, 60%, 80%). The female set contained the 0% (i.e., the original female A), 20% (adding 20% of female B’s face to female A’s face), 40%, 60%, and 80% morphing faces. Likewise, the male set contained the 0% (the original male C), 20% (adding 20% of male D’s face to male C’s face), 40%, 60%, and 80% morphing faces. All face images were sized 13.5 cm (height) by 9.5 cm (width). The task contained 32 trials presented in random order. Female participants received female stimuli while male participants received male stimuli condition. Each trial began with a fixation, then a *target face* was presented for 2 seconds, and a *comparison face* appeared after 1-second blank. Participants were asked to judge whether the two faces look different. The 32 trials consist of 16 “same” trials (0%) and 16 “different” trials (20%, 40%, 60%, or 80% morph). The comparison face remained on the screen until the participant orally answered. The experimenter assisted in making a key-press response, then the next trial began. To make sure the participants understand the task, the experimenter repeated the instructions.

#### Task 2: Dynamic facial emotion recognition

We used neutral, Anger, Disgust, Fear, Happy, Sad, and Surprise expressions for the female and male stimuli. All face images were 17.3 cm (height) by 15.2 cm (width). A total of 12 color dynamic facial emotion GIF videos were created by morphing the neutral face (0% intensity) with the six basic emotions (Anger, Disgust, Fear, Happy, Sad, or Surprise—100% intensity) for each gender. The task contained 24 trials (6 emotions × 2 genders × 2 repetitions) presented in random order. Each trial began with a 1-second blank; then a dynamic facial emotion from neutral to the most intense expression was automatically played for 2 seconds. Participants were told to answer orally at any time when they recognized the emotion. If participants could not answer or take longer than a minute, the experimenter recited the six basic emotions to remind them. Once the participant answered, the experimenter pressed a key to record the response time, wrote down the answer, and proceeded to the next trial. The intention of having the experimenter made the keypress for participants was because it would be much harder for PD-D patients to engage in pushing the key-press due to resting tremors (typically seen in the hand). Therefore, to be fair to all participants, the same experimenter also made the keypress for the control group.

#### Task 3: Expression imitation

We used static facial images expressing the six basic emotions (anger, disgust, fear, happy, sad, and surprise) and their verbal labels (in Chinese characters). The face images were in frontal view, sized 14 cm × 11 cm. We used PowerPoint to create an imitation trial slide show that a verbal label appeared first followed by the corresponding emotional face image. We then converted the slide show to a video (1 minute 40 seconds) containing six imitation trials. The first trial began with a verbal label of “Happy (開心)” for 6 seconds, then a static “Happy” face appeared for 12 seconds for the participants to imitate the “Happy” expression. During the 12 seconds, participants were asked to imitate and hold their expression, or rest after they have done their best imitation; then proceeded to the next trial. This pattern repeated with five other expressions (in the order of Anger, Sadness, Fear, Surprise, and Disgust). Participants’ imitations were recorded live via the laptop camera. Each participant’s imitation videos were analyzed by iMotion Affectiva software–a computer-based automated facial expression analysis that captures raw emotions. The software detects facial landmark, and classify facial expression in return with numeric scores for facial expressions, Action Units (AU) codes, and other metrics. The scores range from 0 (no expression) to 100 (expression fully present).

## Data Availability

The datasets generated and analysed during the current study are not publicly available because it is an on-going double-blinded project, but are available from the corresponding author on reasonable request.
